# Turn the beat around: Commentary on “Slow and fast beat sequences are represented differently through space**"** (De Tommaso & Prpic, 2020, in Attention, Perception, & Psychophysics)

**DOI:** 10.3758/s13414-021-02247-8

**Published:** 2021-03-08

**Authors:** Danielle Wood, Samuel Shaki, Martin H. Fischer

**Affiliations:** 1 Department of Psychology, University of Potsdam, Karl-Liebknecht-Strasse 24-25 House 14, D-14476 Potsdam, OT Golm Germany; 2 Ariel University, Ariel, Israel

**Keywords:** Distance effect, Music cognition, Pitch, magnitude association, Semantic congruity effect, SMARC, Sound recognition, Spatial cognition

## Abstract

There has been increasing interest in the spatial mapping of various perceptual and cognitive magnitudes, such as expanding the spatial-numerical association of response codes (SNARC) effect into domains outside of numerical cognition. Recently, De Tommaso and Prpic (*Attention, Perception, & Psychophysics, 82,* 2765–2773, 2020) reported in this journal that only fast tempos over 104 beats per minute have spatial associations, with more right-sided associations and faster responses for faster tempos. After discussing the role of perceived loudness and possible response strategies, we propose and recommend methodological improvements for further research.

Research into the cognitive association between space and magnitudes has yielded many intriguing findings. The original study (Dehaene, Bossini, & Giraux, 1993) reported faster left-side responses for small digits (such as 1 or 2) and faster right-side responses for larger digits (such as 8 or 9) in a speeded parity classification task. This performance signature was termed the spatial-numerical association of response codes (SNARC) effect and has been replicated and extended to other magnitude-related tasks and stimulus sets (for reviews, see Shaki & Fischer, 2014; Toomarian & Hubbard, 2018).

Recently, De Tommaso and Prpic (2020) reported a SNARC-like effect for auditorily presented musical tempos. Their participants classified target beat sequences as slower or faster than a reference sequence played before, using left-side and right-side response buttons. After conducting three experiments, the authors found a spatial association for fast tempos ranging from 133 to 201 bpm in Experiment 3. However, they did not find this spatial association in the first two experiments, with Experiment 1 evaluating a full range tempo from 40 to 200 bpm and Experiment 2 evaluating slow tempos ranging from 40 to 104 bpm. Based on Experiment 3 and the results of Prpic, Fumarola, De Tommaso, Baldassi, and Agostini (2013), the authors concluded that slow and fast tempos might be differently represented in our minds and that only fast tempos over 104 bpm might have spatial associations, with more right-sided associations for faster tempos.

This study deals with an interesting topic because most of the auditory SNARC-like effects were documented for pitch height (e.g., Fischer, Riello, Giordano, & Rusconi, 2013; Lachmair et al., 2017; Prpic & Domijan, 2018; Rusconi, Kwan, Giordano, Umiltà, & Butterworth, 2006) or temporal duration (Conson et al., 2008; Ishihara, Keller, Rossetti, & Prinz, 2008; Vallesi, Binns, & Shallice, 2008). However, we believe that the conclusions of the current study may be premature because of both methodological questions and conceptual concerns, which we will now discuss in turn.

Considering first a methodological question, neither Prpic et al. (2013) nor De Tommaso and Prpic (2020) specified the perceived pitch height (related to the fundamental frequency of the sound), the exact frequency (usually given in Hz), or perceived loudness (related to the amplitude of stimuli and usually given in dB) of stimuli. They rather stated that the beats had a “metronome-like timbre” and that the amplitude was “set at a comfortable level for each participant and held constant.” (De Tomasso & Prpic 2020, p.2767). To be clear, there is no concern about a methodological confound here because parameters were held constant across conditions; however, the lack of information about either objective intensities or subjective sensations makes replications difficult. This holds true because spatial associations are known to depend on specific values of perceived pitch (i.e., spatial-music association of response codes-effect [SMARC]; De Tommaso & Prpic, 2020), the frequency used, or the perceived loudness. We are therefore grateful that the authors now provide their materials at this link: https://osf.io/gts83/?view_only=a2ad625966e04afd861bef29018b35cc.

Several studies illustrate the possibility of extraneous perceptual or motor biases: In regard to pitch height (and its related frequencies), higher pitch is perceived as faster and lower pitch is perceived as slower (Boltz, 2011; Broze & Huron, 2013; Collier & Hubbard, 2001). Additionally, Varlet, Williams, and Keller (2020) recently found that lower-pitched rhythms had an influence on motor processes, as revealed by participants’ movement entrainment while listening to differently pitched metronomes. When listening to a low-pitched metronome (100 Hz) compared with a high-pitched metronome (1600 Hz), participants moved more consistently in time with the lower-pitched than the higher-pitched metronome. This influence of pitch was also seen by Hove, Marie, Bruce, and Trainor (2014), who reported a low-pitch superiority for timing information during their finger-tapping experiment.

Secondly, perceived loudness can affect participants’ responses because it becomes associated with response side (Chang & Cho, 2015). Possible mechanisms for such associations include a spatially ordered representation of all quantities (e.g., mental number line; Fischer & Shaki, 2014) or a polarity correspondence. According to the polarity correspondence principle (Proctor & Cho, 2006), the association occurs when the stimulus alternatives and the response alternatives are both coded such that one of each receives a “+” and the other a “−” polarity. The result is a congruency effect between loudness and response sides, such that “loud” and “right” become associated (both are “+” poles), while “soft” and “left” become associated as “−” poles (Chang & Cho, 2015; see also Guilbert, 2020). The missing loudness information is a more general issue in the literature on music perception.

Additionally, when a sound intensity of stimuli is the same while their frequencies differ, this will result in different perceived loudness. To minimize this effect, stimuli can be normalized or equalized for perceived loudness, using, for example, Cool Edit Pro software (Hove et al., 2014) or the Cambridge loudness model (Varlet et al., 2020).

Furthermore, De Tommaso and Prpic’s (2020) finding of the spatial association for the narrow stimulus range (fast tempos; Experiment 3), but not for the full range (both fast and slow tempos; Experiment 1) is counterintuitive because the two poles of a continuum should be even more salient on the wider compared with the narrower stimulus continuum. Evidence for this was reported in previous studies that found SNARC effects in both full and partial stimulus ranges (e.g., Antoine & Gevers, 2016; Dehaene et al., 1993; Fias, Brysbaert, Geypens, & d'Ydewalle, 1996). Although it is possible that acoustic rate judgments may behave differently from other modalities and paradigms, this is unlikely given the wide evidence of range independence of polarity correspondence effects across materials and domains. Examples include polarity correspondence between loudness and lateralized response sets (Chang & Cho, 2015), the semantic congruity effect (e.g., Howard, 1983, with humans; Jones et al., 2010, with animals), the symbolic distance effect (e.g., Moyer & Bayer, 1976), and comparative judgments (e.g., Petrusic, 2001). It is worth acknowledging that the authors themselves noticed this challenge and wrote, “In the present study, it is not possible to define the exact moment at which our stimuli were presented, since music tempo is perceived through time and cannot be captured in one precise moment—this is different in comparison to what occurs for numbers and many other kinds of stimuli” (De Tommaso & Prpic, 2020, p. 8).

This brings us to our conceptual concerns. As indicated by the much slower reaction times for Experiment 2 (see Fig. 3 of De Tommaso & Prpic, 2020), slow tempos were slower to discriminate than fast tempos (Experiment 3), which is an apparent violation of the well-established psychophysical Weber–Fechner law. Note that although absolute temporal distances between tempo sequences and reference stimulus were fixed between experiments, their ratio was less than half in Experiment 3 compared with those of Experiment 2. How is it possible that participants spent approximately 50% more time to respond to large versus small tempo ratios?

One possible answer to this conundrum, already alluded to by the original authors, is revealed by close inspection of the reaction times participants produced to respond to the individual tempo sequences: As the authors themselves calculated, participants responded to slower beat sequences before even hearing the second tempo-defining beat for 40 bpm and well before the third beat for 56 bpm. Without this second or third beat, participants cannot have made comparative tempo judgments and rather based their decisions on the duration of the interbeat interval. Moreover, because tempo refers to the rate at which musical notes are played, determining the tempo should rely on the sequence of beats rather than the duration between beats. In other words, while participants were required to discriminate between slow and fast tempos, they may in fact have turned the beat around and decided whether interval durations were shorter or longer than the reference. Related to task difficulty, yet another possible answer is to consider the principle of inverse effectiveness (Holmes, 2009; Stein & Meredith, 1993, p. 143): The more difficult a stimulus is to process, the more information from another modality is used, even when task irrelevant. This means that spatial associations become more important when discrimination is difficult, as for fast compared with slow tempos.^1^

Duration and tempo are negatively correlated: Slower tempo beats create longer interbeat intervals and longer durations. Thus, our interpretation of De Tommaso and Prpic’s (2020) data in terms of a strategic turn can explain the observed absence of overt spatial associations for slow beats as reflecting the presence of a covert conflict: slow beats probably induced *both* a right-side association for long durations (cf. Conson et al., 2008; Vallesi et al., 2008) and a left-side association for slow tempos (consistent with the authors’ remaining data) that cancelled each other. Consistent with this, Ishihara et al. (2008) found that left responses were faster for early onset timing, while right responses were faster for later onset timing. Cancellation of conflicting spatial associations was previously discussed for numerical magnitudes, both in Hebrew readers (Shaki & Fischer, 2012, 2014) and for crossed versus uncrossed hands (Fischer, 2006). De Tommaso and Prpic (2020) already hinted at this possibility when they wrote that there are “two speculations about the focus of participants’ judgements, namely, whether their decision is based on tempo or on time duration” (p. 8). Our reaction-time analysis provides evidence for the presence of these two different strategies, while the error analysis suggests a possible trade-off that should be studied in future.

Future studies of spatial associations for tempo should, for example, turn the beat around and play the target tempo before the reference tempo to ensure complete encoding of the target. Of course, in both versions there is the problem of anticipatory responding, so memory-based judgment might be a useful additional test. Similarly, changing the instructions to either focus on the temporal gap or on the second beat might inform about the role of strategy use. Moreover, implicit tempo evaluation tasks might be useful, such as timbre judgments (cf. Li, 2020; Lidji, Kolinsky, Lochy, & Morais, 2007; Pantev, Roberts, Schulz, Engelien, & Ross, 2001). Reporting perceived loudness would be a welcome addition for reasons elaborated above and can be a component included in future music perception methodology. Secondly, since the error pattern in a task could also reflect a speed–accuracy trade-off (note the somewhat higher accuracy in Experiment 2 compared with the other experiments), both speed and accuracy should be reported.

Alternatively, researchers could use other procedures, maybe without a reference, to evaluate whether the spatial association occurs regardless of whether tempo is relevant or not (e.g., in parity classification, to assess the mental number line). To illustrate, consider two procedures that either remove space from the assessment or instead introduce space to the assessment of spatial associations for the stimulus of interest. By presenting stimuli centrally and also recording responses centrally, we (Fischer & Shaki, 2016; Shaki & Fischer, 2018) established a purely conceptual association between numbers and space that was not contaminated by lateralized spatial task ingredients. Instead, by presenting the stimulus and also collecting its perceptual evaluation in spatially distributed locations, we (Shaki & Fischer, 2020) recently documented the inevitability of space-based distortions in perceptual judgments. The latter work used a production method that also avoids the additional complication of measuring the perception of temporally extended stimuli with temporally extended response measures (i.e., reaction times) by collecting instead perceptual judgments. Clearly, there is a wide range of opportunities to replicate and extend the work reported by Prpic et al. (2013) and De Tommaso and Prpic (2020).

## Footnotes

1 We thank Dr. Valter Prpic and an anonymous reviewer for mentioning these points.

## References

[CR1] Antoine, S., & Gevers, W. (2016). Beyond left and right: Automaticity and flexibility of number-space associations. *Psychonomic Bulletin & Review*, *23*(1). 10.3758/s13423-015-0856-x 10.3758/s13423-015-0856-x 25968089

[CR2] Boltz MG (2011). Illusory tempo changes due to musical characteristics. Music Perception.

[CR3] Broze Y, Huron D (2013). Is higher music faster? Pitch–speed relationships in western compositions. Music Perception: An Interdisciplinary Journal.

[CR4] Chang S, Cho YS (2015). Polarity correspondence effect between loudness and lateralized response set. Frontiers in Psychology.

[CR5] Collier W, Hubbard T (2001). Musical scales and evaluations of happiness and awkwardness: Effects of pitch, direction, and scale mode. The American Journal of Psychology.

[CR6] Conson M, Sacco S, Sarà M, Pistoia F, Grossi D, Trojano L (2008). Selective motor imagery defect in patients with locked-in syndrome. Neuropsychologia.

[CR7] Dehaene S, Bossini S, Giraux P (1993). The mental representation of parity and number magnitude. Journal of Experimental Psychology.

[CR8] De Tommaso M, Prpic V (2020). Slow and fast beat sequences are represented differently through space. Attention, Perception, & Psychophysics.

[CR9] Fias, W., Brysbaert, M., Geypens, F., & d'Ydewalle, G. (1996). The importance of magnitude information in numerical processing: Evidence from the SNARC effect. *Mathematical Cognition*, *2*(1). 10.1080/135467996387552.

[CR10] Fischer MH (2006). The future for SNARC could be stark. Cortex.

[CR11] Fischer, M. H., Riello, M., Giordano, B. L., & Rusconi, E. (2013). Singing numbers . . . in cognitive space. *Topics in Cognitive Sciences*, *5*(2), 354–366. 10.1111/tops.12017 10.1111/tops.12017 23495123

[CR12] Fischer MH, Shaki S (2014). Spatial associations in numerical cognition: From single digits to arithmetic. Quarterly Journal of Experimental Psychology.

[CR13] Fischer MH, Shaki S (2016). Measuring spatial-numerical associations: Evidence for a purely conceptual link. Psychological Research.

[CR14] Guilbert, A. (2020). About the existence of a horizontal mental pitch line in non-musicians. *Laterality: Asymmetries of Body, Brain and Cognition, 25*(2), 215–228. 10.1080/1357650X.2019.1646756 10.1080/1357650X.2019.1646756 31378139

[CR15] Holmes NP (2009). The principle of inverse effectiveness in multisensory integration: Some statistical considerations. Brain Topography.

[CR16] Hove MJ, Marie C, Bruce IC, Trainor LJ (2014). Superior time perception for low musical pitch. Proceedings of the National Academy of Sciences of the United States of America.

[CR17] Howard RW (1983). The semantic congruity effect: Some tests of the expectancy hypothesis. Acta Psychologica.

[CR18] Ishihara M, Keller PE, Rossetti E, Prinz W (2008). Horizontal spatial representation of time: Evidence for the STEARC effect. Cortex.

[CR19] Jones, S. M., Cantlon, J. F., Merritt, D. J., & Brannon, E. M. (2010). Context affects the numerical semantic congruity effect in rhesus monkeys (Macaca mulatta). *Behavioural Processes, 83*(2), 191–196. 10.1016/j.beproc.2009.12.009 10.1016/j.beproc.2009.12.009 PMC3677752 20015467

[CR20] Lachmair M, Cress U, Fissler T, Kurek S, Leininger J, Nuerk H (2017). Music-space associations are grounded, embodied and situated: Examination of cello experts and non-musicians in a standard tone discrimination task. Psychological Research.

[CR21] Li, X. (2020). The effects of timbre on absolute pitch judgment. *Psychology of Music*. 10.1177/0305735619893437

[CR22] Lidji, P. & Kolinsky, R. & Lochy, A. & Morais, J. (2007). Spatial associations for musical stimuli: A piano in the head? *Journal of Experimental Psychology: Human Perception and Performance, 33,* 1189–207. 10.1037/0096-1523.33.5.1189 10.1037/0096-1523.33.5.1189 17924817

[CR23] Moyer RS, Bayer RH (1976). Mental comparison and the symbolic distance effect. Cognitive Psychology.

[CR24] Pantev C, Roberts LE, Schulz M, Engelien A, Ross B (2001). Timbre-specific enhancement of auditory cortical representations in musicians. NeuroReport.

[CR25] Petrusic WM, Sommerfeldt E (2001). Contextual effects and associative processes in comparative judgements with symbolic and perceptual stimuli. *Fechner Day 2001: Proceedings of the Sixteenth Annual Meeting of the International Society for Psychophysics*.

[CR26] Proctor RW, Cho YS (2006). Polarity correspondence: A general principle for performance of speeded binary classification tasks. Psychological Bulletin.

[CR27] Prpic V, Domijan D (2018). Linear representation of pitch height in the SMARC effect. Psihologijske Teme.

[CR28] Prpic V, Fumarola A, De Tommaso M, Baldassi G, Agostini T (2013). A SNARC-like effect for music tempo. Review of Psychology.

[CR29] Rusconi E, Kwan B, Giordano BL, Umiltà C, Butterworth B (2006). Spatial representation of pitch height: The SMARC effect. Cognition.

[CR30] Shaki S, Fischer MH (2012). Multiple spatial mappings in numerical cognition. Journal of Experimental Psychology: Human Perception and Performance.

[CR31] Shaki S, Fischer MH (2014). Removing spatial responses reveals spatial concepts—Even in a culture with mixed reading habits. Frontiers in Human Neuroscience.

[CR32] Shaki S, Fischer MH (2018). Deconstructing spatial-numerical associations. Cognition.

[CR33] Shaki, S., & Fischer, M. H. (2020). Systematic spatial distortion of quantitative estimates. *Psychological Research*10.1007/s00426-020-01390-5 10.1007/s00426-020-01390-5 PMC8357716 32676794

[CR34] Stein, B. E., & Meredith, M. A. (1993). *The merging of the senses.* MIT Press.

[CR35] Toomarian, E. Y., & Hubbard, E. M. (2018). On the genesis of spatial-numerical associations: Evolutionary and cultural factors co-construct the mental number line. *Neuroscience and Biobehavioral Reviews*, *90*, 184–199. 10.1016/j.neubiorev.2018.04.010 10.1016/j.neubiorev.2018.04.010 PMC5993626 29684402

[CR36] Vallesi A, Binns M, Shallice T (2008). An effect of spatial-temporal association of response codes: Understanding the cognitive representations of time. Cognition.

[CR37] Varlet M, Williams R, Keller PE (2020). Effects of pitch and tempo of auditory rhythms on spontaneous movement entrainment and stabilisation. Psychological Research.

